# Editorial: The next phase of public health: innovations from the private sector to build health equity, collaborations, and resilience

**DOI:** 10.3389/frhs.2023.1297266

**Published:** 2023-12-14

**Authors:** Susan Garfield, Amy White, Frances Garfield, Richard Siegrist, Natasha Eslami

**Affiliations:** ^1^ Ernst and Young, New York, NY, United States; ^2^ Lehman College, Bronx, NY, United States; ^3^ Independent Researcher, Cambridge, MA, United States; ^4^ School of Public Health, Harvard University, Boston, MA, United States

**Keywords:** public health, health equity, collaboration, resilience, private sector

**Editorial on the Research Topic** The next phase of public health: innovations from the private sector to build health equity, collaborations, and resilience

The Covid-19 pandemic highlighted foundational challenges in managing the public's health and the persistent health inequities in our communities. Within the private sector, companies witnessed how adverse health events impact the economy at large, their employees, and their customers. As a result, many companies have begun to see that they have a critical role in improving the long-term well-being of the communities in which they work and how health equity can build resilience. This recognition has driven many to action. Either on their own or in collaboration with established public health players, companies are deploying private sector points of view and practices to public health and health equity challenges.

With investments in innovation and collaboration, companies are contributing expertise, technology, and capability to advance patient experience and outcomes while attempting to improve resilience to public health events in an unknown future. They bring resources, perspectives, and approaches that are often distinct from how Public Health practitioners, policymakers, and community organization have worked to date. As such, collaborations can bring together complementary resources and expertise to address challenges each organization couldn't solve on its own. For example, companies can support public organizations by providing technical expertise, financial support, and education, as well as a platform for positive publicity and exposure. Meanwhile, public health entities can provide specialized knowledge, pre-existing community capital, and lessons learned from prior interventions. Though challenges exist, leveraging complementary skills to synergistically impact Public Health and Health Equity challenges holds significant promise.

In this Research Topic of Frontiers in Health Services, titled *The Next Phase of Public Health: Innovations from the Private Sector to Build Health Equity, Collaborations, and Resilience*, the contributing authors explore *How Best to Collaborate*, the *Challenges of Innovation Readiness*, and *Drivers of Success*. Through observational studies and review articles, the authors examine best practices from the private sector, perspectives on evaluating organizational readiness and resilience, and ways to overcome collaboration challenges. Together, they challenge stakeholders within and beyond the private sector to operate more effectively as equity and resilience change agents on their own and in collaboration with others.

## How best to collaborate

Once organizations understand their own resources, goals, and capabilities, collaboration opportunities emerge. But the path to successful collaborations to advance Health Equity is challenging. In their paper discussing the collaborative assessment of online women's health education tools, Edouard et al. discuss elements of a successful collaboration between an academic institution and a corporate partner. They review how to first align corporate and academic purposes and then how critical to success communication, alignment of goals, and shared decision-making are throughout. Taylor et al. used multiple retrospective case studies to examine how business and nonprofits build sustainable partnerships. They found that partnerships that acknowledged differences were more successful and resilient. Arnaout et al. report four key enablers for enduring and financially successful partnership models. They describe how one major technology company's efforts to increase access to health services not only allowed underserved communities to be served during or after a crisis but also created a foundation for the impacted communities to build the needed knowledge, capacity, and resources to tackle unexpected future crises. Partnerships like this rely on functional organizational and operational models to work, often different from either group's individual structure. Aveling et al. discuss the benefits of emerging hybrid organizing models, the forms hybrid organizations can take, and how each can work over time to advance collaborative public health and health equity goals. In addition, Ingman et al. demonstrate the power of partnerships in advancing evidence-based practice in childhood obesity. They found that effective partnerships, a nuanced approach to fidelity, scalability considerations, and the role of technical assistance and training all contributed to the successful implementation of their local public health agency - elementary school partnership.

## Challenges of innovation readiness

To understand organizations' readiness for and likely resilience when faced with various public health threats, Garfield et al. developed their Public Health Resilience Assessment Tool which explores how companies and other organizations are positioned to respond. It also highlights areas of needed growth and underscores what resources can be leveraged to advance an organization's own or collaborative goals related to Public Health. The paper then examines the tools' application to 8 companies across sectors. Pradier et al. discuss a model for addressing complex health challenges at the community level through ongoing exchange of information and engagement as experienced through the Open Arena for Public Health. They highlighted challenges to changes and innovations challenges through building collective intelligence and conducting ongoing policy dialogues.

## Drivers of success

Bringing innovations from the private sector to advance Health Equity and Public Health involves understanding critical success factors like goal clarity, transparency, and data sharing. Cronin and Franz discuss the public availability of private hospitals' Community Benefits and Implementation Reports as part of the private sector's collaboration with the public health system. Arnaout et al. build on these insights by highlighting the key role of bidirectional data exchange in creating successful public-private partnerships. Data can be leveraged to support the case for change and demonstrate clinical and economic benefits on both sides. The authors also highlight how emerging technologies such as AI can unlock additional value by drawing further insights that lead to more targeted interventions.

As more private sector programs in Public Health and Health Equity are launched, people may move out of the public sector into these new opportunities. This mobility and cross-pollination of skills and experience is largely positive but may also have unforeseen negative outcomes. White’s article on *Transitioning from Medicaid to Private Health Insurance* showed that on the individual level, employees moving to the private sector need education and support from their employers throughout the transition process to address core issues like health insurance continuity.

Overall, the researchers in this Research Topic demonstrate that there are multiple factors critical to successfully harnessing private sector participation in addressing Public Health and Health Equity challenges. The private sector's speed, resourcing, and organizational focus can accelerate the impact of their interventions. However, given that collaboration is critical to most endeavors, understanding how to best set up private-public partnerships, work together, and leverage data and technology to advance common goals will be essential for success.

